# Supplementary material for: Temporal Relationships between Smartphone Application Use and Online Substance Procurement in U.S. Youth
Source: medRxiv. 2026 May 19:2026.05.15.26353324. Preprint. [Version 1] doi: 10.64898/2026.05.15.26353324 (PMC13228776; doi:10.64898/2026.05.15.26353324)
Supplement: 1 [file NIHPP2026.05.15.26353324V1-supplement-1.pdf]

medRxiv preprint doi: https://doi.org/10.64898/2026.05.15.26353324; this version posted May 19, 2026. The copyright holder for this preprint (which was not certified by peer review) is the author/funder, who has granted medRxiv a license to display the preprint in perpetuity.
It is made available under a CC-BY 4.0 International license .

SUPPLEMENTARY TABLE 1: OUTCOME QUESTIONS
**1). Contact via social media about procuring substances**
TODAY, did anyone contact you ONLINE or over SOCIAL MEDIA about purchasing or accessing drugs or alcohol?<br>• Yes<br>• No
**2) Contact via direct message about procuring substances**
TODAY, did anyone contact you through a DIRECT MESSAGE or TEXT about purchasing or accessing drugs or alcohol?<br>• Yes<br>• No
**3) Obtained substances that day (any method of procurement)**
TODAY, did you obtain drugs or alcohol in any way?<br>• Yes<br>• No
**4) Obtained substances that day using social media**
If yes to question #3:<br>TODAY, did you purchase or obtain drugs or alcohol using a social media platform?<br>• Yes<br>• No
